# System Framework of Robotics in Upper Limb Rehabilitation on Poststroke Motor Recovery

**DOI:** 10.1155/2018/6737056

**Published:** 2018-12-13

**Authors:** Kai Zhang, Xiaofeng Chen, Fei Liu, Haili Tang, Jing Wang, Weina Wen

**Affiliations:** ^1^Institute of Robotics and Intelligent System, School of Mechanical Engineering, Xi'an Jiaotong University, Xi'an 710049, China; ^2^Shaanxi Key Laboratory of Intelligent Robots, Xi'an 710049, China; ^3^Baoxing Hospital, Shenzhen 518100, China

## Abstract

Neurological impairments such as stroke cause damage to the functional mobility of survivors and affect their ability to perform activities of daily living. Recently, robotic treatment for upper limb stroke rehabilitation has received significant attention because it can provide high-intensity and repetitive movement therapy. In this review, the current status of upper limb rehabilitation robots is explored. Firstly, an overview of mechanical design of robotics for upper-limb rehabilitation and clinical effects of part robots are provided. Then, the comparisons of human-machine interactions, control strategies, driving modes, and training modes are described. Finally, the development and the possible future directions of the upper limb rehabilitation robot are discussed.

## 1. Introduction

Stroke is one of the leading causes for disability. In China, there are more than 2 million new cases every year. More than 1.5 million people die from stroke each year, and three quarters of the survivors have varying degrees of sequelae [1]. The weakness and loss of the control of the upper limb that arise from nerve damage are the main symptoms [2]. This disease not only brings pain and heavy financial burden to patients and families but also brings huge economic losses and some social problems to the country.

With the development of robot technology, the application of robot in rehabilitation has aroused wide concern in the international community. A series of intelligent rehabilitation robots including artificial prosthesis and external mechanical auxiliary system have successfully developed to help patients to achieve functional recovery or compensation for the loss of motor function [3–5].

**Figure 1:**
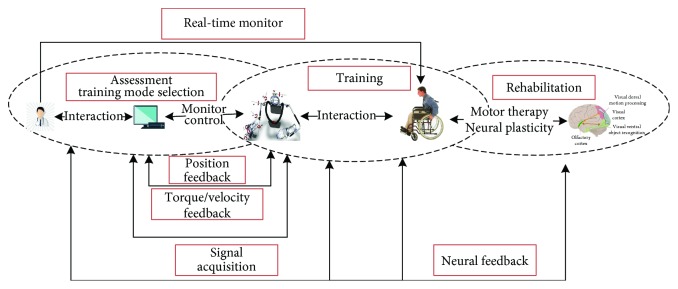
Upper-limb rehabilitation robot system.

There are two types of rehabilitation robot for upper extremity: one is end-effector upper limb rehabilitation robots, another is exoskeleton rehabilitation robot [6]. These robots can provide rehabilitation training tasks used to guide the patients to complete targeted rehabilitation training (Figure 1). At the same time, the provision of repetitive and intensive physical therapy greatly reduces the burden of physical therapists [7–10].

This paper gives a systematic review of research status in an upper limb rehabilitation robot. In order to review the development of the robot in more detail, we divide following chapters to present. Firstly, we provide a classification of typical mechanisms and clinical effects of part robots. The next section introduces the comparisons of human-machine interactions, control strategies, driving modes, and training modes. Then, the third section gives an overview of development and the possible future directions of the upper limb rehabilitation robot.

## 2. Method

A wide literature search from 1985 until December 2017 has been conducted resorting to the main databases, such as Web of Science, Google Scholar, and IEEE Xplore databases. The keywords used for the electronic search were upper limb, exoskeleton, end-effector type, and rehabilitation robotics. The studies that satisfied these criteria were (1) technology of part of the rehabilitation robot system, (2) robot that is able to assist the stroke patients to exercise upper limb movement, (3) focus on upper-limb functional abilities, and (4) control strategy and man-machine interaction. A total of 230 papers have been gathered by using the aforementioned search method; 117 papers have been excluded since they did not meet the inclusion criteria.

## 3. Classification of Typical Mechanisms and Evolution of Upper Limb Robot

The rehabilitation training robot was used to assist patients to complete the rehabilitation training. It has different design requirements of general industrial robots because of special working objects and environment. Usually, rehabilitation robots can be divided into end-effector type and exoskeleton according to the robot's different ways of supporting and pulling the limb. Specifically, the end-effector rehabilitation robot can be divided into rigid rod traction type and rope traction type [11, 12]. Compared with the rigid rod traction, the rope traction can realize the passive motion training and the active training of the more complex trajectory in the plane. The exoskeleton robot is installed outside the body called wearable robot [13–15]. The joints and linkages of the exoskeleton have a direct correspondence with the human body, and it requires the robot joint rotation center consistent with the anatomical axis of the human body [15–17].

### 3.1. End-Effector Rehabilitation Robot

The end-effector rehabilitation robot system consists of ordinary connecting rod and series robot mechanism. In the working state, the robot drives the movement of the upper limbs by connecting with the patient's arm to achieve the rehabilitation training. The robot system is relatively independent of the patient, which only connects to the end of the robot.

The upper limb rehabilitation robot firstly appeared in 1993. Lum et al. developed a “hand-object-hand” system used in the upper limbs of hemiplegic stroke patients after a period of time of rehabilitation [18]. The patient's hands are at two clamp plate handles. The curve or extension movement of the wrist with the help of a drive motor can only be achieved.

In 1995, Lum et al. developed a hand-lifting recovery machine (bimanual lifting rehabilitator) to train patients with both hands [19]. It requires patients to lift the object and move the action. The system can assist patients by completing the lift and movement in the same hand when the hand cannot produce enough force to move. Three hemiplegic stroke patients participated in the experiment (lift the handles). The ADLS test indicated that one patient has greatly improved the state of therapy.

Since 1995, Krebs et al. of the United States have developed MIT rehabilitation robots and expanded their function. So far, the mechanical parts of the robot have three modules: graphic module, wrist module, and hand module. The robot assists patients to complete the drawing of the elbow, forearm, and wrist joint movement and hand grasping training. Twenty hemiparetic stroke patients were divided into a control group and an experimental group in the clinical trial. The latter were required to execute traction movement of the shoulder and elbow with a machine. Results indicated that patients in the experimental group improved further and faster (Fugl-Meyer (*P* ≤ 0.20) and motor power scores (*P* ≤ 0.10), motor status score (*P* ≤ 0.05)) [20].

In 2000, Stanford University invented a robot named MIME upper limb rehabilitation robot. It can help patients to complete the upper limb rehabilitation training in a mirror image of the contralateral movement. Through the acquisition of the contralateral movement and the industrial robot assistant, patients completed the mirror rehabilitation training. Two subacute stroke patients accepted the treatment during three weeks. Fugl-Meyer, Box and Block Test, and Jebsen-Taylor Test proved the improvement on the patients' upper limb especially the finger and hand [21, 22].

In 2002, the British Reading University developed an upper limb rehabilitation robot named GENTLES. It is the first time to apply virtual reality technology in a rehabilitation robot. Patients can complete the rehabilitation training independently by gravity compensation function and the visual feedback. Eight healthy subjects participated in the experiment. They feedback that this system is more suitable for those stroke patients with some athletic abilities [23, 24].

Since 2004, the research achievements of upper limb rehabilitation robots have mushroomed, and various kinds of rehabilitation robots have appeared. In our country, although rehabilitation medicine engineering attracts wide attention, rehabilitation robot research is still in its infancy stage. In Tsinghua University, researchers have carried out the auxiliary neurological rehabilitation research, and they have successfully developed a rehabilitation robot with complex movement which satisfied the training requirement of the shoulder, elbow, and hand [25, 26].

### 3.2. Exoskeleton Rehabilitation Robot

Using end-effector upper limb rehabilitation robots can complete the complex trajectory of rehabilitation training exercise, but it is difficult to achieve the accurate movement of the concrete joints. Therefore, there is another kind of upper limb rehabilitation robot system—the exoskeleton of the upper limb rehabilitation robot system available.

The exoskeleton robot is a wearable device that combines mechanical power device and intelligence control strategy. Structurally, exoskeleton robots can be divided into upper limb exoskeleton robot, lower limb exoskeleton robot, whole body exoskeleton robot, and all kinds of joint correction or restorative training skeletal robot. The exoskeleton rehabilitation robot provides power compensation, body protection, and support. It integrated sensor and control information, which can coordinate with the patient to complete the auxiliary training of body movements.

Southampton University has developed the famous 5-DOF SAIL upper limb rehabilitation robot without power source. It equipped revolute joints with a torsional spring elasticity auxiliary support system in the shoulder and elbow. It combined the virtual reality (VR) technology with electrical stimulation technology to complete the training on the shoulder, elbow, and wrist, achieving good healing properties. Eleven healthy subjects and five stroke patients participated in the experiment. The results confirmed that striking improvement has been seen for the anterior deltoid [27].

The Arizona University at United States developed 5-DOF upper limb rehabilitation robot RUPERT and artificial 4-DOF robot driven by pneumatic muscle (PM). Movement driven by the pneumatic muscle is more flexible, because of similar muscle function and movement characteristics. The modified Wolf Motor Test and the Fugl-Meyer upper motor assessment test were utilized. Eight healthy subjects and two stroke patients participated in this experiment. Four healthy subjects improved in both the simple and complex tasks, and two patients improved the limb function [28–30].

Perry from the University of Washington developed a 7-DOF arm rehabilitation robot named CADEN-77 driven by rope besides forearm rotation [31, 32]. It can complete flexion and stretch of the shoulder, rotation of the arm, flexion and extension of the elbow, and flexion, stretch, outreach, and adduction of the wrist. In this system, most of the actuators and moderators were equipped in the shoulder in order to realize long-distance transmission and simplify the structure of the robot. In addition, it sharply reduced the impact of gear drive and friction. Because of the existence of multidegrees of freedom, each movement of the robot is more precise. The clinical study indicated that it can realize the entire arm, shoulder, elbow, and wrist parts of the rehabilitation training and achieve good effect. However, one degree of freedom of movement of joint actually is divided into two motions: reverse direction and positive direction. In order to complete the movement, there are two ropes working, respectively. What is more, rope in sports is always in a state of tension in order to ensure the continuity of movement back and forth. Therefore, achieving the motion requirements needs a complex winding device. Due to the intrinsic characteristics of cable transmission, it is prone to producing elastic sliding. Therefore, robot movement is not accurate. Yu and Rosen developed a rehabilitation-training exobiology-UL7 system at the same time based on this robot, each arm driven by a CADEN-7 machine [33].

The University of California and the University of Irvine developed 5-DOF T-WREX (training Wilmington robotic exoskeleton) [34, 35] and 5-DOF Pneu-WREX upper limb rehabilitation robot systems [36] used to train the shoulder and elbow. The former is suitable for active training and movement parameter measurement for patients without drive. The latter adopts pneumatic drive, which is further suitable for patients with passive rehabilitation training. Both can achieve flexion and stretch of shoulder and elbow. A drive cylinder can realize the dynamic balance, which can reduce the gravity of the robot movement. Spring also is used to support gravity. Five patients with chronic stroke participated in this study. Results show that gravity balance training improved reaching ability to the contralateral target but not to the ipsilateral target and improved the vertical reaching range of motion.

Rice University developed a composite group organization 5-DOF forearm rehabilitation robot named MAHI Exo II [37]. The first half of the robot was a 3-DOF parallel mechanism that can realize wrist bend, stretch, adduction, extension, and complete arm stretch. The second half was a 2-DOF serial mechanism. It can achieve forearm rotation and elbow bend and stretch. Also, elbow rotation was placed with a counterweight to overcome the arm weight.

Northwestern University in collaboration with the Rehabilitation Institute of Chicago studied a 10-DOF (8 + 2) of the upper limb rehabilitation robot named IntelliArm [38]. It can realize the movement of the whole upper limb, including flexion and stretch, inside rotary and outside rotary, outreach and adduction, and grasp and put. Besides achieving active movement, it also can realize passive movement with 2-DOF. Compared to the traditional robotic, IntelliArm could also provide more accurate and quantitative diagnosis in clinical practice.

Queen's University designed the 6-DOF upper limb rehabilitation robot MEDARM driven by a rope [39]. Sternoclavicular joints have two degrees of freedom, and shoulder joints have three degrees of freedom; elbow joints have one degree of freedom. The robot can contact the shoulder and elbow to complete the composite movement. As well as CADEN-7 and MEDARM, the transmission system is too complex. This robot can accommodate users of varying shape and size and have the potential to be widely used in clinic.

Canada's ETS cooperation with McGill University has developed a 7-DOF arm rehabilitation robot system named MARSE ETS [40–42]. It can realize flexion and stretch of the shoulder, inside and outside movement of the rotary, rotation of the arm, flexion and extension of the elbow, and flexion, stretch, outreach, and adduction of the wrist. The system chooses Maxon motor as power source, which can complete the whole upper limb joint rehabilitation training.

The Swiss Royal Institute of Technology cooperation with the affiliated hospital of Balgrist University developed the famous 6-DOF arm rehabilitation robot ARMin [43]. It can realize the up and down movement of the whole robot system, inward and outward rotation of the shoulder, rotation of the arm, and flexion and stretch of the wrist. It also can realize 2-DOF passive movements which are flexion and stretch of the shoulder and rotation of the forearm. Then they cooperated with Ljubljana University of Slovenia which developed the ARMin II upper limb rehabilitation robot [44, 45]. The robot is a total of seven degrees of freedom machine; it can realize the up and down movement of the whole robot system, flexion and stretch, inward and outward rotation of the shoulder, rotation of the forearm, and flexion and stretch of the wrist and elbow. In addition, ARMin II provides gravity compensation with limbs and assists the limb of the shoulder joint and elbow in complex movement. As power source, DC motors provide power to ensure machine normal movement.

In summary, the exoskeleton robot can realize more accurately the assist motion, and the end-effector rehabilitation robot has better performance on feedback and evaluation. The exoskeleton has parallel motion range and space with human joints, so it can reduce inertia more effectively and have a more compact structure. However, the end-effector is a series mechanism, which has larger movement space and more freedom. The end-effector is more suitable for multifreedom training tasks. Besides, the end-effector can effectively solve the joint coincidence problem, but underactuated exoskeleton can also realize it. Exoskeletons are increasingly becoming a major option in clinical treatment compared to the end-effector.

The overview of end-effector upper limb rehabilitation robots and exoskeleton upper limb rehabilitation robots is shown in Tables 1 and 2.

## 4. Human-Machine Interaction

Human-machine interaction is an important part of the robot system, which can influence the recovery effect and treatment process largely. This paper argues that human-machine interaction refers to communication methods, which are distinguished by the type of control signal. From this point of view, human-machine interaction can divide into bioelectric signal interaction method and physical signal interaction method.

### 4.1. Bioelectric Signal Interaction

#### 4.1.1. Brain Computer Interface Interaction

BCI technology is one of research hotspots in recent years that formed in the 1970s [46]. The principle of brain-computer interface technology can explain as follows.

**Figure 2:**
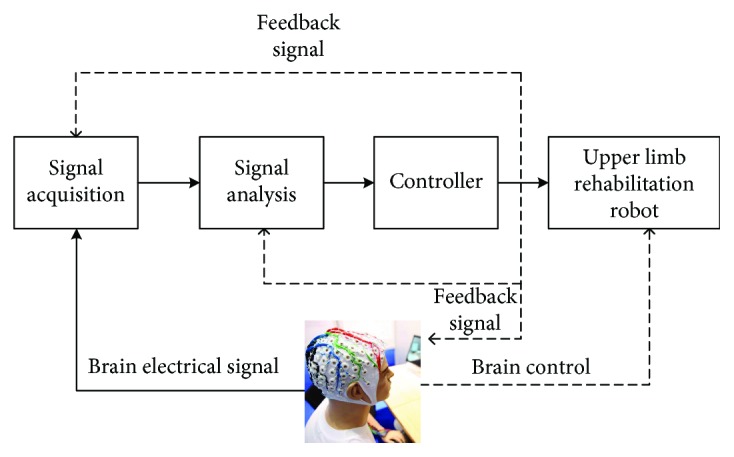
Block diagram of brain machine interface system.

When the brain is doing conceptual work, creating motion mind, or receiving outside stimulation, the nerve cells will produce dozens of millivolts of microelectrical activity. The electrical activity of a large number of nerve cells transmitted to the surface of the scalp to form brainwaves. This EEG (electroencephalograph) will reflect some characteristics of rhythm and spatial distribution and can be detected by a certain method. By signal processing, the intention signal of humans is discriminated and converted into a control command to realize controlling of external equipment and communication with the outside [47].  Figure 2 shows the principle of the brain machine interface system.

The two kinds of signal acquisition methods commonly used in the BCI system are intrusive and noninvasive. The signal collected by the equipment is amplified by amplifiers and pretreated by the processor, which is converted into digital signals and is stored in the computer. In BCI, the following signals are often collected: visual evoked potential (VEP), event-related potential (ERP) P300, slow cortical potential (SCP), spontaneous EEG Alpha wave, and Mu and beta rhythm signal.

With the development of neuroscience, scientists found that in the whole process of life, the function of the damaged central nervous system can restore through the action of reasonable physiological potential [48–50]. We can combine the BCI system with upper limb rehabilitation robots to realize the reconstruction of the upper limb movement function. A study proved that the BCI system combined with prosthesis movements in time could lead to motor learning and induce neural plasticity or neural compensation, which induced motor function improvement [51].

#### 4.1.2. Surface Muscle Electrical Signal Interaction

The surface electromyogram signal produced by the contraction of the muscle is obtained by the surface electrode from the muscle surface of the human body. The electrical control system obtains multichannel sEMG from the human skin surface by the electrode and then performs eigenvalue extraction and action discrimination. The processor determines the motion state of the corresponding joints and muscles according to the identification results. And then it can control peripheral devices.

Researches show that in the process of joint movement, sEMG can not only reflect the muscle information such as fatigue state and contraction intensity but also reflect movement intentions and other information in the process of different body joints movement [52].

The identification of motor intention relies heavily on the selection of the signal acquisition site. After extracting and processing the EMG signal, the upper limb rehabilitation robot estimates joint torque and designs moving targets, then the processor controls the robot to complete the corresponding movement according to the instruction. Niigata University developed the upper limb rehabilitation robot based on the electromyogram signal control. The robot consists of seven DC motors, encoder, point meter, and the force and torque sensors, which can assist patients to complete seven degrees of freedom movement and training [53].

### 4.2. Motion Parameter Feedback Interaction

Compared with bioelectric signals, the motion parameters' interactive signals have advantages of accuracy and reliability. The ways of motion parameters are force control, position control, and force/position hybrid control. Force sensors in real time, which are processed, judged, and translated into instructions to control the machine, often collect signals. Similarly, the variation of the position signal is used as the control signal. These different kinds of way of interaction often use the impedance control algorithm, PID control algorithm, and trajectory tracking control algorithm.

In recent years, the interactive mode of motion parameters are often combined with virtual reality technology and applied in the field of motion rehabilitation. It shows that using virtual reality (VR) and computer game techniques in post-stroke upper limb rehabilitation may enhance neuronal plasticity [54–57]. Recent studies indicate that brain damage can be improved by a highly repetitive and task-oriented training, and this response can be optimized if the task is challenging enough [58, 59]. Multichannel sensory feedback is key in reestablishing the neural pathways damaged by stroke and closing the sensor motor loop [60]. The rehabilitation robot platform combined with VR (virtual reality) and the RGS (rehabilitation gaming system) gives a possible reestablishment of the damaged motor cortex which can be activated with the mediation of mirror neurons or through the patient's motor imagery. The traditional rehabilitation robots based on virtual reality technology apply to the patients with acute or subacute stroke stage, but recent studies have shown that patients with chronic stroke patients can effectively recover by the technology of the robot platform combined with VR (virtual reality) and the RGS (rehabilitation gaming system). A five-degree-of-freedom hand rehabilitation robotic device named Amadeo was used as the experimental platform, which can provide position based on passive and active assistive training modes that emphasize the flexion and extension of each finger. A chronic stroke subject who underwent the proposed rehabilitation approach showed improvement in clinical evaluation methods using the assessment methods (Fugl-Meyer Assessment, Motor Assessment Scale, and Range of Motion) [61].

In summary, the interaction mode based on bioelectric signal has better effects on nerve rehabilitation treatment and has greater clinical application potential. The physical signal interaction method is more suitable for patients with some motion capability and has more potential in the application of family rehabilitation. Bioelectric signal interaction can express the patient's needs and physical condition at the neural level, especially brain signal interaction which has crucial significance in clinical neurological rehabilitation. Though physical signals are more stable and accurate, they cannot better represent people's intentions. In the future, the rehabilitation robot will be controlled based on the brain signals combined with physical feedback and virtual reality environment so that the user's neural activity can be better activated.

## 5. Control Strategies of Upper Limb Rehabilitation Robots

In recent reviews, many researchers summarized human-machine interaction, training modes, and control strategies into one type to categorize and compare (Table 3). This paper divided three parts described above. HMI has been presented in the previous part. This section will focus on introducing control strategies, and the next section will describe training modes.

Different control strategies of robot can be realized by the processing of human-machine interaction signals in different methods and algorithms. The control strategy can be divided into following parts.

### 5.1. Position Control

The position control is also known as trajectory tracking control. The angular displacement of each joint is determined by the kinematic inversion of the planned trajectory that controls the torque output of the motor in the process of computer operation to drive the motion of each joint and realize the movement along the planned trajectory [68].

### 5.2. Force Control

Each joint moment sensor captures the motion of the upper limbs of hemiplegic stroke patients in real time. The control system can convert joint torque to the equivalent force of the end effector according to the characteristics of institutions. Then, the controller can drive the motor to achieve the multijoint motion of the upper limb according to this equivalent force. Some researchers have also validated the clinical efficacy of force feedback. Johnson et al. designed the experiment to research the effect of using force-feedback control in robot-assisted stroke therapy [69].

### 5.3. Force/Position Hybrid Control

Researchers put forward that independently the control force and position at the same time are the optimal schemes of the robot control method. In theory, the free force space and position free space of the robot is two complementary orthogonal subspaces; the parameters are independently controlled in each space. The constraint environment is considered as a geometric problem with no deformation at this time [70–74].

### 5.4. Impedance Control

Impedance control is proposed by Hogan, who points out that the relationship between the position and the force can be described by a generalized nonlinear impedance model with a property of inertia, damping, or stiffness [75, 76].

The relationship between speed and force is called mechanical impedance. The objective of impedance control is to mediate the mechanical impedance of the robot to maintain the ideal dynamic relationship of the contact force and position between the end-effector and the environment [77]. Therefore, robots based on impedance strategy can provide a comfortable and soft touch for patients. Akdoğan et al. developed a complete rehabilitation system consisting of a human machine interface and a hybrid impedance controller that is aimed at completing therapeutic exercises for wrist and forearm rehabilitation [78]^.^

In a word, position control can effectively control joint angles and provide real-time feedback. For different patients, position control can make the training more personalized. Force control requires accurate acquisition of muscle and machine interactions so it relies more on the layout of the sensor. Impedance focuses on realizing the flexibility of the rehabilitation robot which avoids excess between mechanical structure and limbs. This method could provide a natural, comfortable, and safe touch interface and avoid secondary damage effectively. In practical applications, force/position hybrid control is a better control method in passive training mode and impedance is more suitable in active training processes.

## 6. Driving Modes of Upper Limb Rehabilitation Robots

There are three common driving modes for upper limb rehabilitation robots: motor drive, hydraulic drive, and pneumatic muscle drive. The advantages and disadvantages of the three driving modes are shown in Table 4.

## 7. Training Modes of Upper Limb Rehabilitation Robots

Robotic intervention therapy for stroke patients shows clear improvement of scores and strength, but these progresses are not reflected daily. It is necessary to focus on the targeted stage of recovery [86]. Therefore, the training mode is also an important factor affecting the rehabilitation effect [87]. A reasonable rehabilitation program is that patients with different degrees of rehabilitation should adopt different training patterns [88]. Brunnstrom put forward six stages of the recovery of limb rehabilitation process, which is divided into flaccid paralysis period, cramps period, and recovery period. In different stages of recovery, the patient's muscle tension will make different changes to transfer limb movement patterns among detached motion mode, common motion mode, and multimodal motion mode [89–93]. The training modes of the upper limb robot can be divided into passive mode and active mode.

### 7.1. Passive Mode

Passive training is almost performed by a physical therapist or by a device. It requires the movement of joint in the regular range and prevents contractures to maintain the static length of the muscles [94]. The passive rehabilitation training can be described that patients follow the robot to complete the scheduled trajectory present by a therapist through interaction. Trajectory tracking and mirroring motion are common methods [95]; proportional-integral-derivative algorithm can be applied in this mode [96–99]. Mirroring motion mode requires uninjured side limb driving injured side limb to complete symmetrical training; researcher conduct clinical trials to verify the effect [100, 101].

### 7.2. Active Mode

There is a limitation of passive training mode because patients cannot make effort to engage training [102]. Two typical active control modes for rehabilitation robots are active assist mode and active resist mode. In the process of active assist training, patients contract muscle and had complete training with the assistance of external force. In the process of power-assisted motion, machines only provide the minimum force that can meet the prescribed motion. The auxiliary force should be adjusted according to the condition of recovery and muscle force to achieve this coordinated movement pattern. Active resist training means that the patient must overcome external resistance to complete the movement during the exercise.

In the active rehabilitation training, the main motion form is autonomous movement of the patient, and the robot can judge the patient's motor ability according to the movement information of the patients to provide appropriate assistance. Active rehabilitation training can be divided into active power mode and active impedance. The robot can judge the sport ability and recovery degree by gathering motion information of the interaction between the robot and the patient and decide to provide assistance or resistance.

## 8. Discussion

In the past few decades, the upper limb robots applied to clinical rehabilitation have brought positive significance to stroke patients. In this paper, the development of upper limb rehabilitation robot, human machine interaction, training mode, driving mode, and control strategy is reviewed. The upper limb rehabilitation robots have shown encouraging clinical outcomes and rehabilitation efficiency. Previous researchers have accomplished a lot of work to promote the development of this technology. However, this study still faces many challenges.

In terms of mechanical structure design, the complexity of the upper limb joints and redundancy DOFs bring many difficulties. The simplified model of arm exoskeleton movement usually may lead to several possible problems. During the training process of physical therapy, the unanticipated force generated during the man-machine collaboration may reduce the comfort level of humans and robot system. It may be better to optimize and improve institutional material and guarantee the flexible contact between patients and robots. Wearable robots often require as little weight as possible, but the simplified model may reduce the working space of the upper limb exoskeleton system thereby limiting its application scope. Most equipment is stationary; the patients cannot flexibly use rehabilitation equipment anytime and anywhere because of the style of installation of equipment. It cannot satisfy the portable, mobile, and other requirements and greatly affect patient rehabilitation experience, so it is necessary to make machine more lightweight and portable in the future study.

Among the upper limb joints, the shoulder joint is a complex joint because the center of rotation is moving with nonlinear trace in the process of movement. Therefore, it is necessary to solve the problem that the rotation center of machine misalignment with the rotation center of the human body in future studies. Compared with end-effector rehabilitation robots, the exoskeleton has better bionic characteristics, because its motion is similar to human joint motion. In the future, the exoskeleton should be more anthropomorphic and the design of motion should be similar to biomechanical joint motion.

From the perspective of the human biomechanics aspect, one of the important features is the physical properties of the musculotendinous and their resulting impedance. Therefore, exoskeletons should accordingly adapt to these variations of impedance to guarantee the smoothness of contacting and preventing spasm. In the process of active control, the exoskeleton mechanism singularity exists in its working space, increasing the difficulty of the control algorithm. It is helpful to reduce the complexity of the control algorithm to avoid the singularity in the actual workspace by optimizing a machine design.

In the process of working conditions, it is essential for a rehabilitation robot to guarantee the patient's safety. Therefore, the robot should guarantee the safety of the subject from the following aspects: using the joint sensor to monitor the force information of the subjects during the movement, when the reaction force caused by the muscle tension is too high. When the patient appears with muscle spasm, the robot should automatically stop the current movement so as not to cause the muscle strain. The working space should be limited in the reasonable range by limiting the switch in order to avoid the repeated injury for the patient. The movement speed and displacement of the robot should also be limited by software. The operator should reasonably set the parameters of the driving device and monitor the robot motion state in real time. A press-and-stop button is extremely important to avoid accident situations. Also, self-locking of robot should be taken into account to avoid the damage by the rotation of the joints caused by gravity.

It has been shown that using virtual reality (VR) and computer game techniques in stroke rehabilitation may enhance the function of neuronal plasticity. Further, enhancing patient sensorial state can be accomplished by merging together with VR, haptic and vibrotactile feedback. However, real-time synchronization of signals that is dedicated to reconstruct VR may be delayed due to the large number of required devices. This may lead to a bad real-time environmental reconstruction, thus increasing the task difficulty. It is also necessary to improve the response speed of the signal to ensure the timeliness of the interaction between the virtual environment and humans.

BCI technology provides a new way of communication and control without language or body movement. It can directly express ideas or manipulate machine by the brain that has profound research significance in neurological rehabilitation. However, there is a difference in physiological characteristics of the brain between patients and normal individuals. Heterogeneity in post-damage expression inevitably complicates the decoding of brain signals responsible for neuronal plasticity recovery; this may lead to complicate extraction of suitable control inputs. The best way is integrating BCIs with actual rehabilitation methods to establish a powerful and accurate rehabilitative scenario. So far, there are BCI systems that hardly exist that can provide a high-accuracy control [64].

It is often thought that the rehabilitation of the proximal arm may benefit the distal arm. As revealed by many studies, the proximal improvements in the arm do not necessarily transfer to the distal arm or vice versa and that arm improvements do not manifest as improved ADL performance. Therefore, how to design a more reasonable device to ensure the best recovery of the proximal and distal end is also a problem that needs to study.

## Figures and Tables

**Table 1:** Overview of End-effector upper limb rehabilitation robots.

Groups	Devices	Researchers	DOFs	Driving modes	Control strategies	Training mode	Experimental subject	Functional testing	Clinical scale
End-effector rehabilitation robot	hand-object - hand	Lum et al. [18]	1	The contralateral hand drive	Force control	Active mode	\	\	\
End-effector rehabilitation robot	Bimanual lifting rehabilitator	Lum et al. [19]	2	Motor drive	Force control	Passive mode	Three hemiplegic patients	Lift handles	ADLS improved
End-effector rehabilitation robot	MIT MANUS	Krebs et al. [20]	3	Motor drive	Impedance control	Active mode Passive mode	Twenty hemiparetic patients	Traction movement	Fugl-Meyer (*P* ≤ 0.20) Motor power scores (*P* ≤ 0.10) Motor status score (*P* ≤ 0.05) improved
End-effector rehabilitation robot	MIME	Stanford university [21]	6	Motor drive	EMG signal control Force control	Active mode Passive mode	Two subacute stroke patients	Finger and hand motion	Fugl-Meyer, Box and Block test, Jebsen-Taylor test improved
End-effector rehabilitation robot	ARM Guide	Chicago institute of rehabilitation [22]	3	Motor drive	Impedance control	Active mode Passive mode	Nineteen hemiparetic patients	Free reaching	Univariate ANOVA statistics; ADLS
End-effector rehabilitation robot	GENTLE/S	University of Reading [23]	3	Motor drive	Position control	Passive mode	Thirty-one patients	\	Multivariate analysis of the Fugl-Meyer improved
End-effector rehabilitation robot	Haptic-robotic exercise platform	Lam et al. [24]	2	Motor drive	Impedance control Position control	Active mode Passive mode	Eight healthy subjects	Traction movement	Quantitative data improved Administered questionnaire improved
End-effector rehabilitation robot	EMUL	Osaka university [103]	6	Hydraulic drive Motor drive	Impedance control	Active mode Passive mode	Five hemiparetic patients	Virtual tasks	Ueda Fugl-Meyer Motoricity Index improved

**Table 2:** Overview of exoskeleton upper limb rehabilitation robots.

Groups	Devices	Researchers	DOFs	Driving modes	Control strategies	Training mode	Experimental subject	Functional testing	Clinical scale
Exoskeleton rehabilitation robot	RUPERT	University of Arizona [28–30]	4	Pneumatic drive	Force control Impedance control	Passive mode	Eight healthy subjects and two stroke patients	Motion tasks	Fugl-Meyer Wolf Motor Test improved
Exoskeleton rehabilitation robot	CADEN-7	University of Washington [31, 32]	7	Motor drive Line drive	Position control	Passive mode	Six subjects	\	ADLS improved
Exoskeleton rehabilitation robot	T-WREX	The university of California [34, 35]	5	Pneumatic drive	Force control	Active mode Passive mode	Five chronic stroke patients	Self-control experiment	Fugl-Meyer improved
Exoskeleton rehabilitation robot	MAHI Exo II	Rice University [37]	5	Motor drive	Position control	Active mode	/	/	/
Exoskeleton rehabilitation robot	IntelliArm	Northwestern University [38]	10	Motor drive	Force/Position control	Active mode Passive mode	/	/	/
Exoskeleton rehabilitation robot	MEDARM	Queen University [39]	6	Line drive	Position control	Passive mode	/	/	/
Exoskeleton rehabilitation robot	MARSE ETS	McGill University [40–42]	7	Motor drive	EMG signal control	Active mode	/	/	/
Exoskeleton rehabilitation robot	ARMin	The royal Swiss institute of technology [43]	6	Motor drive	Force control Impedance control	Active mode Passive mode	A healthy subject	Traction movement	/
Exoskeleton rehabilitation robot	ARMin II	The royal Swiss Institute of Technology [44, 45]	7	Motor drive	Force control	Active mode Passive mode	Eight hemiplegic Three incomplete spinal cord injured subjects	Virtual tasks	ADLS improved

**Table 3:** The classification of control strategy.

Term	Description	Standard of classification
Maciejasz et al. [62]	**“High-level” control strategy** (i) Assistive control (ii) Challenge-based control (iii) Haptic stimulation (iv) Couching control	The function of control systems
	**“Low-level” control strategy** (i) Admittance control (ii) Impedance control	

Gopura et al. [63]	**Based on input information** (i) Human biological signal based control methods (ii) Nonbiological signal-based control methods (iii) Platform-independent control methods	The position of signals
	**Based on output information** (i) Different controller architecture	

Anam et al. [64]	**Model system** (i) Dynamic (ii) Muscle	Control system architectures
	**Physical parameters** (i) Position (ii) Torque/force (iii) Force interaction	
	**The hierarchy** (i) Task level (ii) High level (iii) Low level	
	**Usage** (i) Virtual reality (ii) Teleoperation (iii) Gait	

Marchal-Crespo et al. [65]	**Assistive controllers** (i) Impedance-based assistance (ii) Counterbalancing assistance (iii) EMG-based assistance (iv) Performance-based adaption of task parameter	Type of human-machine interaction
	**Challenge-based robotic therapy control algorithms** (i) Resistive strategies (ii) Constraint-induced strategies (iii) Error-amplification strategies	
	**Haptic simulation strategies** (i) Virtual reality	
	**Noncontacting coaches** (i) Noncontacting coaches	

Sicur et al. [66]	Passive Active unassisted Active assisted Resistive	Training modes

Proietti et al. [67]	**Assistive mode** Passive control Triggered passive control Partially assistive control **Corrective mode** Tunneling Coordination control **Resistive mode**	Training modes

**Table 4:** Advantages and disadvantages of the three driving modes.

	Motor drive [79, 80]	Hydraulic drive [81, 82]	Pneumatic muscle drive [83–85]
Advantage	(i) The cable for connection has advantages of energy transfer convenient, signal transform quickly (ii) High level standard (iii) Easily to achieve automatic control (iv) Simple structure (v) Nonpolluting	(i) High reliability (ii) Simple structure (iii) Low inertia (iv) The overload protection is easily realized (v) It can realize stepless speed regulation (vi) Working stability	(i) Simple structure (ii) Low cost (iii) Small gas viscosity (iv) It can realize stepless speed regulation (v) Nonpolluting (vi) Little resistance losing (vii) Fire and explosion prevention, high flow rate (viii) Working in high temperature

Disadvantage	(i) It has poor balance of movement (ii) It's easily influenced by external load (iii) Large inertia (iv) Slow change (v) Large volume (vi) Heavy	(i) It is sensitive to oil temperature and loading change; (ii) The hydraulic oil can be compressed; (iii) The working fluid is easy to leak (iv) High noise (v) Low energy efficiency (vi) Low drive speed	(i) The gas is easy to be compressed and leak (ii) The speed is easy to change under the load (iii) It is difficult to precise control cannot be used under low temperature (iv) The gas is difficult to sealed (v) Working pressure is usually smaller than 0.8 MPa, which only applies to small power driving (vi) Unsuitable for a high-power system

## References

[1] Wang W. (2009). Chinese stroke epidemiology and community groups to intervene. *Chinese Journal of the Frontiers of Medical Science (Electronic Version)*.

[2] Zeferino S. I., Aycock D. M. (2010). Poststroke shoulder pain: inevitable or preventable?. *Rehabilitation Nursing*.

[3] Zhang X. Y., Wang K. X. (2013). Robot assisted rehabilitation technology, robotics and intelligent devices. *Chinese Journal of Rehabilitation*.

[4] Tan M., Wang S. (2013). Research progress on robotics. *Acta Automatica Sinica*.

[5] Jamwal P. K., Hussain S., Ghayesh M. H., Rogozina S. V. (2016). Impedance control of an intrinsically compliant parallel ankle rehabilitation robot. *IEEE Transactions on Industrial Electronics*.

[6] Gopura R. A. R. C., Kiguchi K. Mechanical designs of active upper-limb exoskeleton robots: state-of-the-art and design difficulties.

[7] Fasoli S. E., Krebs H. I., Stein J., Frontera W. R., Hogan N. (2003). Effects of robotic therapy on motor impairment and recovery in chronic stroke. *Archives of Physical Medicine and Rehabilitation*.

[8] Riener R., Nef T., Colombo G. (2005). Robot-aided neurorehabilitation of the upper extremities. *Medical & Biological Engineering & Computing*.

[9] Colombo R., Sterpi I., Mazzone A., Delconte C., Pisano F. (2015). Improving proprioceptive deficits after stroke through robot-assisted training of the upper limb: a pilot case report study. *Neurocase*.

[10] Norouzi-Gheidari N., Archambault P. S., Fung J. (2012). Effects of robot-assisted therapy on stroke rehabilitation in upper limbs: systematic review and meta-analysis of the literature. *The Journal of Rehabilitation Research and Development*.

[11] Enchen L., Manan L. (2014). Advance in upper limb rehabilitation robot (review). *Chinese Journal of Rehabilitation Theory and Practice*.

[12] Rosati G., Zanotto D., Secoli R., Rossi A. Design and control of two planar cable-driven robots for upper-limb neurorehabilitation.

[13] Gupta A. (2004). Design and control of a haptic arm exoskeleton.

[14] Gupta A., O'Malley M. K. (2006). Design of a haptic arm exoskeleton for training and rehabilitation. *IEEE/ASME Transactions on Mechatronics*.

[15] Pehlivan A. U., Rose C., O'Malley M. K. System characterization of RiceWrist-S: a forearm-wrist exoskeleton for upper extremity rehabilitation.

[16] Ragonesi D., Agrawal S., Sample W., Rahman T. Series elastic actuator control of a powered exoskeleton.

[17] Martinez J. A., Ng P., Lu S., Campagna M. K. S., Celik O. Design of wrist gimbal: a forearm and wrist exoskeleton for stroke rehabilitation.

[18] Lum P. S., Reinkensmeyer D. J., Lehman S. L., Li P. Y., Stark L. W. (1992). Feedforward stabilization in a bimanual unloading task. *Experimental Brain Research*.

[19] Lum S. P., Lehman S. L., Reinkensmeyer D. J. (1995). The bimanual lifting rehabilitator: an adaptive machine for therapy of stroke patients. *IEEE Transactions on Rehabilitation Engineering*.

[20] Krebs H. I., Hogan N., Aisen M. L., Volpe B. T. (1998). Robot-aided neurorehabilitation. *IEEE Transactions on Rehabilitation Engineering*.

[21] Daud O. A., Oboe R., Agostini M., Turolla A. Performance evaluation of a VR-based hand and finger rehabilitation program.

[22] Kahn L. E., Zygman M. L., Rymer W. Z., Reinkensmeyer D. J. (2006). Robot-assisted reaching exercise promotes arm movement recovery in chronic hemiparetic stroke: a randomized controlled pilot study. *Journal of Neuroengineering and Rehabilitation*.

[23] Amirabdollahian F., Loureiro R., Gradwell E., Collin C., Harwin W., Johnson G. (2007). Multivariate analysis of the Fugl-Meyer outcome measures assessing the effectiveness of GENTLE/S robot-mediated stroke therapy. *Journal of NeuroEngineering and Rehabilitation*.

[24] Lam P., Hebert D., Boger J. (2008). A haptic-robotic platform for upper-limb reaching stroke therapy: preliminary design and evaluation results. *Journal of NeuroEngineering and Rehabilitation*.

[25] Adamovich S. V., Merians A. S., Boian R. (2005). A virtual reality—based exercise system for hand rehabilitation post-stroke. *Presence: Teleoperators and Virtual Environments*.

[26] Teodorescu H.-N., Jain L. (2000). *Intelligent Systems and Technologies in Rehabilitation Engineering*.

[27] Cai Z., Tong D., Meadmore K. L. Design & control of a 3D stroke rehabilitation platform.

[28] Sugar T. G., Jiping He, Koeneman E. J. (2007). Design and control of RUPERT: a device for robotic upper extremity repetitive therapy. *IEEE Transactions on Neural Systems and Rehabilitation Engineering*.

[29] Balasubramanian S., Wei R., Perez M. RUPERT: an exoskeleton robot for assisting rehabilitation of arm functions.

[30] Zhang H., Austin H., Buchanan S., Herman R., Koeneman J., He J. Feasibility study of robot-assisted stroke rehabilitation at home using RUPERT.

[31] Perry J. C., Rosen J. Design of a 7 degree-of-freedom upper-limb powered exoskeleton.

[32] Perry J. C., Rosen J., Burns S. (2007). Upper-Limb Powered Exoskeleton Design. *IEEE/ASME Transactions on Mechatronics*.

[33] Yu W., Rosen J. A novel linear PID controller for an upper limb exoskeleton.

[34] Sanchez R., Reinkensmeyer D., Shah P. Monitoring functional arm movement for home-based therapy after stroke.

[35] Sanchez R. J., Wolbrecht E., Smith R. A pneumatic robot for re-training arm movement after stroke: rationale and mechanical design.

[36] Rosen J., Perry J. C., Manning N., Burns S., Hannaford B. The human arm kinematics and dynamics during daily activities - toward a 7 DOF upper limb powered exoskeleton.

[37] Pehlivan A. U., Celik O., O'Malley M. K. Mechanical design of a distal arm exoskeleton for stroke and spinal cord injury rehabilitation.

[38] Ren Y., Park H. S., Zhang L. Q. Developing a whole-arm exoskeleton robot with hand opening and closing mechanism for upper limb stroke rehabilitation.

[39] Ball S. J., Brown I. E., Scott S. H. MEDARM: a rehabilitation robot with 5DOF at the shoulder complex.

[40] Rahman M. H., Ouimet T. K., Saad M., Kenne J. P., Archambault P. S. Development and control of a wearable robot for rehabilitation of elbow and shoulder joint movements.

[41] Kiguchi K., Hayashi Y. (2012). An EMG-based control for an upper-limb power-assist exoskeleton robot. *IEEE Transactions on Systems, Man, and Cybernetics, Part B (Cybernetics)*.

[42] Lo H. S., Xie S. Q. (2012). Exoskeleton robots for upper-limb rehabilitation: state of the art and future prospects. *Medical Engineering & Physics*.

[43] Nef T., Riener R. ARMin - design of a novel arm rehabilitation robot.

[44] Nef T., Mihelj M., Riener R. (2007). ARMin: a robot for patient-cooperative arm therapy. *Medical & Biological Engineering & Computing*.

[45] Mihelj M., Nef T., Riener R. ARMin II -7 DoF rehabilitation robot: mechanics and kinematics.

[46] McFarland D. J., Wolpaw J. R. (2011). Brain-computer Interfaces for Communication and Control. *Communications of the ACM*.

[47] Blankertz B., Dornhege G., Schafer C. (2003). Boosting bit rates and error detection for the classification of fast-paced motor commands based on single-trial EEG analysis. *IEEE Transactions on Neural Systems and Rehabilitation Engineering*.

[48] Schwartz A. B., Cui X. T., Weber D. J., Moran D. W. (2006). Brain-controlled interfaces: movement restoration with neural prosthetics. *Neuron*.

[49] Daly J. J., Wolpaw J. R. (2008). Brain-computer interfaces in neurological rehabilitation. *The Lancet Neurology*.

[50] Scherberger H. (2009). Neural control of motor prostheses. *Current Opinion in Neurobiology*.

[51] Dayan E., Cohen L. G. (2011). Neuroplasticity subserving motor skill learning. *Neuron*.

[52] Sun Q. Z., Sun Y. N., Ding X. F. (2004). Development and implementation of athlete′s training monitor and analysis system based on surface electromyography. *Journal of Biomedical Engineering Research*.

[53] Kiguchi K. (2007). Active exoskeletons for upper-limb motion assist. *International Journal of Humanoid Robotics*.

[54] Laver K. E., George S., Thomas S., Deutsch J. E., Crotty M. (2010). Virtual reality for stroke rehabilitation. *The Cochrane Library*.

[55] Bardorfer A., Munih M., Zupan A., Primozic A. (2001). Upper limb motion analysis using haptic interface. *IEEE/ASME Transactions on Mechatronics*.

[56] Burdea G., Popescu V., Hentz V., Colbert K. (2000). Virtual reality-based orthopedic telerehabilitation. *IEEE Transactions on Rehabilitation Engineering*.

[57] Mihelj M., Novak D., Milavec M., Ziherl J., Olenšek A., Munih M. (2012). Virtual rehabilitation environment using principles of intrinsic motivation and game design. *Presence: Teleoperators and Virtual Environments*.

[58] Stewart K. C., Cauraugh J. H., Summers J. J. (2006). Bilateral movement training and stroke rehabilitation: a systematic review and meta-analysis. *Journal of the Neurological Sciences*.

[59] Cauraugh J. H., Lodha N., Naik S. K., Summers J. J. (2010). Bilateral movement training and stroke motor recovery progress: a structured review and meta-analysis. *Human Movement Science*.

[60] Poling G. L., Weisenberger J. M., Kerwin T. The role of multisensory feedback in haptic surface perception.

[61] Huang X., Naghdy F., Naghdy G., du H., Todd C. (2018). The combined effects of adaptive control and virtual reality on robot-assisted fine hand motion rehabilitation in chronic stroke patients: a case study. *Journal of Stroke and Cerebrovascular Diseases*.

[62] Maciejasz P., Eschweiler J., Gerlach-Hahn K., Jansen-Troy A., Leonhardt S. (2014). A survey on robotic devices for upper limb rehabilitation. *Journal of NeuroEngineering and Rehabilitation*.

[63] Gopura R. A. R. C., Bandara D. S. V., Kiguchi K., Mann G. K. I. (2016). Developments in hardware systems of active upper-limb exoskeleton robots: a review. *Robotics and Autonomous Systems*.

[64] Anam K., Al-Jumaily A. A. (2012). Active exoskeleton control systems: state of the art. *Procedia Engineering*.

[65] Marchal-Crespo L., Reinkensmeyer D. J. (2009). Review of control strategies for robotic movement training after neurologic injury. *Journal of NeuroEngineering and Rehabilitation*.

[66] Sicuri C., Porcellini G., Merolla G. (2014). Robotics in shoulder rehabilitation. *Muscles, Ligaments and Tendons Journal*.

[67] Proietti T., Crocher V., Roby-Brami A., Jarrasse N. (2016). Upper-limb robotic exoskeletons for neurorehabilitation: a review on control strategies. *IEEE Reviews in Biomedical Engineering*.

[68] Gang Y. U., Qian J. W., Shen L. Y. (2013). Control system design for upper limb rehabilitation robot. *Machinery Design & Manufacture*.

[69] Johnson M. J., VanderLoos H. F. M., Burgar C. G., Shor P., Leifer L. J. (2005). Experimental results using force-feedback cueing in robot-assisted stroke therapy. *IEEE Transactions on Neural Systems and Rehabilitation Engineering*.

[70] Zhang H., Paul R. Hybrid control of robot manipulators.

[71] Paul R. P. Problems and research issues associated with the hybrid control of force and displacement.

[72] Craig J. J. (1986). Introduction to robotics: mechanics and control. *Addison-Wesley Publishing Company*.

[73] Whitney D. Historical perspective and state of the art in robot force control.

[74] Aktan M. E., Akdoğan E. (2018). Design and control of a diagnosis and treatment aimed robotic platform for wrist and forearm rehabilitation: DIAGNOBOT. *Advances in Mechanical Engineering*.

[75] Hogan N. Impedance control: an approach to manipulation.

[76] Hogan N. (1985). Impedance control: an approach to manipulation: part III—applications. *Journal of Dynamic Systems, Measurement, and Control*.

[77] Chen F., Fei Y. Q., Zhao X. F. (2005). The impedance control method for robots. *Modular Machine Tool & Automatic Manufacturing Technique*.

[78] Akdoğan E., Aktan M. E., Koru A. T., Selçuk Arslan M., Atlıhan M., Kuran B. (2018). Hybrid impedance control of a robot manipulator for wrist and forearm rehabilitation: performance analysis and clinical results. *Mechatronics*.

[79] Deluca C., Erim Z. (1994). Common drive of motor units in regulation of muscle force. *Trends in Neurosciences*.

[80] Erim Z., Beg M. F., Burke D. T., de Luca C. J. (1999). Effects of aging on motor-unit control properties. *Journal of Neurophysiology*.

[81] Meier S., Reisch T. (2011). *Hydraulic drive*.

[82] Otten A., Voort C., Stienen A., Aarts R., van Asseldonk E., van der Kooij H. (2015). LIMPACT: a hydraulically powered self-aligning upper limb exoskeleton. *IEEE/ASME Transactions on Mechatronics*.

[83] Jiang X., Xiong C., Sun R., Xiong Y. Fuzzy hybrid force-position control for the robotic arm of an upper limb rehabilitation robot powered by pneumatic muscles.

[84] Wu J., Wang Y., Huang J., Huo W. (2011). Novel wearable multi-DOF upper limb rehabilitation robot driven by pneumatic muscle. *Journal of Huazhong University of Science and Technology*.

[85] Deaconescu A. (2017). Pneumatic muscle actuated rehabilitation equipment of the upper limb joints. *IOP Conference Series: Materials Science and Engineering*.

[86] Basteris A., Nijenhuis S. M., Stienen A. H. A., Buurke J. H., Prange G. B., Amirabdollahian F. (2014). Training modalities in robot-mediated upper limb rehabilitation in stroke: a framework for classification based on a systematic review. *Journal of NeuroEngineering and Rehabilitation*.

[87] Sellin A., Niglas A., Õunapuu-Pikas E., Kupper P. (2014). Rapid and long-term effects of water deficit on gas exchange and hydraulic conductance of silver birch trees grown under varying atmospheric humidity. *BMC Plant Biology*.

[88] Schmidt H., Werner C., Bernhardt R., Hesse S., Krüger J. (2007). Gait rehabilitation machines based on programmable footplates. *Journal of NeuroEngineering and Rehabilitation*.

[89] Brunnstrom S. (1970). Movement therapy in hemiplegia. *A Neurophysiological Approach*.

[90] Melchiorre P. J. (1996). Brunnstrom’s clinical kinesiology. *American Journal of Physical Medicine & Rehabilitation*.

[91] Sawner K., Lavigne J. M., Brunnstrom S. (1992). *Brunnstrom's movement therapy in hemiplegia: a neurophysiological approach*.

[92] Brunnström H., Gustafson L., Passant U., Englund E. (2009). Prevalence of dementia subtypes: a 30-year retrospective survey of neuropathological reports. *Archives of Gerontology and Geriatrics*.

[93] Wagenaar R. C., Meijer O. G., Kuik D. J. (1990). The functional recovery of stroke: a comparison between neuro-developmental treatment and the Brunnstrom method. *Scandinavian Journal of Rehabilitation Medicine*.

[94] Toth A. Passive robotic movement therapy of the spastic hemiparetic arm with REHAROB: report of the first clinical test and the follow-up system improvement.

[95] Proietti T., Jarrassé N., Roby-Brami A., Morel G. Adaptive control of a robotic exoskeleton for neurorehabilitation.

[96] Garrido J., Yu W., Soria A. Modular design and modeling of an upper limb exoskeleton.

[97] Lin C. L., Jan H. Y., Shieh N. C. (2003). GA-based multiobjective PID control for a linear brushless DC motor. *IEEE/ASME Transactions on Mechatronics*.

[98] Kelly R. (1997). PD control with desired gravity compensation of robotic manipulators a review. *The International Journal of Robotics Research*.

[99] Moubarak S., Pham M. T., Moreau R., Redarce T. Gravity compensation of an upper extremity exoskeleton.

[100] Pignolo L. Upper limb rehabilitation after stroke: ARAMIS a “robo-mechatronic” innovative approach and prototype.

[101] Mayr A., Kofler M., Saltuari L. (2008). ARMOR: Elektromechanischer Roboter für das Bewegungstraining der oberen Extremität nach Schlaganfall. Prospektive randomisierte kontrollierte Pilotstudie. *Handchirurgie, Mikrochirurgie, Plastische Chirurgie*.

[102] Lynch D., Ferraro M., Krol J., Trudell C. M., Christos P., Volpe B. T. (2005). Continuous passive motion improves shoulder joint integrity following stroke. *Clinical Rehabilitation*.

[103] Haraguchi M., Kikuchi T., Jin Y. 3-D rehabilitation systems for upper limbs using ER actuators/brakes with high safety: "EMUL", "Robotherapist" and "PLEMO".

